# A Sierra Leone 2021 Midwifery Clinical Training Needs Assessment: A Call to Action to Augment Clinical Precepting

**DOI:** 10.5334/aogh.3970

**Published:** 2023-02-13

**Authors:** Mustapha Sonnie, Frederica Kella, Amy Stern, Clelia Anna Mannino, Sara Adelman, Laura Fuller, Leigh Forbush, Julie Mann, Brittney van de Water, Bryn Falahee, Sadath Sayeed, Helen Ewing, Vanessa Kerry

**Affiliations:** 1 Seed Global Health, SL; 2 Seed Global Health, US

**Keywords:** midwifery, clinical, training, Sierra Leone, needs assessment

## Abstract

**Objective:**

Sierra Leone has one of the highest maternal mortality and infant mortality rates globally. We share findings from a Midwifery Clinical Training Needs Assessment, conducted in 2021 as a collaboration between the Government of Sierra Leone and Seed Global Health. The assessment identified existing needs and gaps in midwifery clinical training at health facilities in Sierra Leone from various stakeholders’ perspectives.

**Methods:**

The descriptive needs assessment utilized mixed methods, including surveys, focus group discussions (FGDs), interviews, and reviews of maternal medical records.

**Results:**

The following showed needs and gaps in labor and delivery management; record keeping; triage processes; clinical education for students, recent graduates, and preceptors; and lack of infrastructure and resources.

**Conclusion:**

The knowledge gained from this needs assessment can further the development of midwifery clinical training programs in Sierra Leone and other low-income countries facing similar challenges. We discuss the implication of our findings.

## Introduction

The commitment to maternal child health is a high priority for Sierra Leone’s government. In 2019, the Office of the Vice President of Sierra Leone invited Seed Global Health (Seed) to partner with the Ministry of Health and Sanitation (MoHS) to support their efforts to decrease preventable maternal mortality through strengthening midwifery training. To inform the best approach to invest in midwifery training, the collaboration sought to elucidate some of the challenges faced in clinical training. In 2021, Seed supported a rapid needs assessment focused on public midwifery training institutions and their associated practice facilities and sought the perspectives of various stakeholders in these settings. This study reports the findings from the needs assessment.

## Background

Sierra Leone (SL) has a population of seven million people, 41% of whom are less than age 15. Twenty-five percent of women are of reproductive age and have an average of four children, and in 2019, 83% of births were delivered at a health facility [1,2]. While SL has done a remarkable job of reducing its maternal mortality rate (MMR) by over fifty percent from 2000 to 2017 and by another fifty percent from 2017 to 2020, it still has the 3rd highest MMR in the world with 510 deaths per 100,000 livebirths [3,4].

While addressing maternal mortality is complex, the global shortage of midwives poses a significant challenge, one particularly acute throughout Africa [5]. Sierra Leone has fewer than 500 midwives, with over 40% working in an urban setting and collectively serving less than 15% of the population [6]. The SL Government projects it needs 3,000 midwives to provide minimally adequate maternal health care services [6]. The Government and Sierra Leone Nurses and Midwives Board recognize the urgency to increase the number of midwives to improve the quality of services [7]. To accomplish this, midwifery education is being strengthened, exemplified through the development of the direct-entry midwifery program. However, there are still factors hampering the quality of clinical education in public midwifery training programs. These include poor collaboration between hospitals and health training institutions, a lack of clinical instructors and preceptors, and a weak framework for assessing students’ clinical performance.

## Methodology

### Study design

With support from the Government, Seed designed the mixed-methods, descriptive needs assessment; and the Institute for Development, a Sierra Leonean consultancy firm, collected the data between April and June 2021. Ethics approval was obtained in SL (Sierra Leone Ethics and Scientific Review Committee, Freetown, Sierra Leone) and the United States (Massachusetts General Hospital, Boston, USA). The assessment focused on the four public midwifery schools: National School of Midwifery, School of Midwifery Makeni, School of Midwifery Bo, and University of Sierra Leone College of Medicine and Health Science.

### Data collection

This assessment utilized surveys, focus group discussions, and interviews with current midwifery students, recent midwifery graduates (graduated within three years), midwifery school staff, and health facility and district health management staff. Participants who volunteered to participate in the needs assessment reported on their clinical rotations and preceptorship, providing insight about their experiences. Data collection tools were developed for each format and participant type, it was important to customize the questions based on the participant type, given the nuances/perspectives from each group.

The team also conducted a facility assessment and maternal patient records review within eight facilities across three districts that sought to understand: 1) how labor and delivery care was managed, 2) health outcomes at the healthcare facilities, and 3) context in which clinical training took place. Maternal patient records were reviewed for all women admitted to the labor ward from either the months between April and June 2021 or up to 100 records. While each record represents one individual patient, data was collected from a variety of sources (i.e., case notes, partographs, and admission, maternity, and delivery registers). The tool for data collection was modified from the World Health Organization (WHO) assessment for emergency obstetric and newborn care (EmONC). Facility visits included an assessment of the use of EmONC protocols, which is an evidence-based strategy used to provide guidance for handling life-threatening complications during pregnancy, childbirth, and the postpartum period [8]. The facility visits also involved an evaluation of availability of a modified form of EmONC protocols in the labor ward, and medicines and consumables for managing postpartum hemorrhage in the hospitals and Community Health Centers. Data collectors counted protocols as available if they were physically present (i.e., laminated charts, wall charts, posters, leaflets) and appropriately placed in a visible location.

### Process for obtaining participants

For students, a handout was developed by the assessment team and an announcement made during classes by the principal/tutors. While the school helped introduce the assessment, it was made explicit that this was not a school survey. For graduates of the programs, the team worked with the school to create a list of midwives who graduated in the last 5 years. From this roster, the team contacted potential participants. For the student and graduate focus groups, purposive selection was used to engage participants with diversity in age, background and experience. Preceptors and heads of maternity were approached with the opportunity to participate.

### Data analysis

Descriptive statistics (frequencies, percentages) were used to analyze the quantitative data. Qualitative data, such as focus group discussions and individual interviews, were analyzed using a grounded theory approach, in which transcripts were examined to find generalizable themes [9]. Those emergent themes were coded with keywords/phrases, and then those codes were grouped into larger concepts/findings.

## Results

### Participants interviews & records review

Participants were from four public midwifery training institutions in SL that offered three-year entry level midwifery diploma programs and included 202 current midwifery students, 74 recent midwifery graduates, 39 preceptors and 8 midwifery school staff. A total of eight district management team sisters and 35 heads of maternity were interviewed from eight district health clinics. Four hundred and eighty-seven clinical records were reviewed from four hospitals and four community health centers.

#### Referral challenges

Delayed referrals from Community Health Centers (CHCs) and peripheral health units (PHUs) to hospitals and late arrival from home deliveries were cited in multiple interviews as causes of poor maternal outcomes. Due to delays from the catchment villages to the first health center, women arriving at the CHC often needed a referral to a hospital with more comprehensive EmONC services. Only 25% (n = 4) of CHCs documented the time between the decision to transfer and the actual transfer. Descriptions of referral criteria varied widely.

#### Triage

There were varying descriptions of the triage process for maternity patients at CHCs and hospitals. Heads of maternity inconsistently described how critical cases were assessed and prioritized upon admission.

#### Vital signs

The patient records review assessed blood pressure (taken every four hours), temperature (taken every two hours), and pulse rate (taken every thirty minutes) as the three vital signs taken during active labor as per protocol. Blood pressure was the most consistently performed vital sign. CHCs were more consistent in completing vital signs with 74% (n = 181) of patients having documented blood pressures as per protocol, compared to 44% (n = 247) in hospitals. Hospitals documented patient temperatures 19% (n = 247) of the time and CHC’s 30% (n = 181) of the time; and patient pulse rates were documented 26% (n = 247) of the time in hospitals and 46% (n = 181) in CHCs.

#### Partograph and documentation

Women in active labor had documentation of a partograph 85% (n = 281) of the time in hospitals and 99% (n = 183) in CHCs. Fourteen percent (n = 251) of women in hospitals had a partograph that showed signs of obstructed labor versus 2% (n = 181) in CHCs. There was no standardization in record keeping between hospitals and CHCs nor between midwives and physicians, and records were paper-based with disparate and incomplete filing systems.

#### Emergency obstetric and newborn care (EmONC) protocols

Out of the 11 EmONC protocols, some were missing from the labor ward in both CHCs and hospitals, including protocols around obstructed labor, incomplete abortion, and manual removal of the placenta and ruptured uterus (Table 1). CHC’s were more likely to have protocols in a visible place.

**Table 1:** EmONC protocol availability on labor ward.


EMONC CONDITION MANAGEMENT	HOSPITAL (N = 4)	COMMUNITY HEALTH CENTER (CHC) (N = 4)

Post-partum-hemorrhage	100%	100%

Ante partum-hemorrhage	75%	100%

Sepsis in newborn	50%	100%

Puerperal sepsis	100%	75%

Pre-eclampsia/eclampsia	100%	100%

Magnesium sulphate use	100%	100%

Neonatal resuscitation	75%	100%

Obstructed labor	50%	50%

Incomplete abortion	25%	50%

Manual removal of placenta	0%	25%

Ruptured uterus	0%	0%


#### Prevalence and management of postpartum hemorrhage at hospitals

Based on the records reviewed, one out of ten (11%) patients in the labor ward at hospitals were diagnosed with postpartum hemorrhage, whereas only 1% of patients at CHCs were. Nearly all women (95%) in the four hospitals and (100%) in the four CHCs where data was collected received oxytocin for active management of the third stage of labor. A variety of interventions were used to treat hospitalized patients diagnosed with postpartum hemorrhage (Table 2). No women with postpartum hemorrhage received bimanual compression or uterine balloon tamponade.

**Table 2:** Hospital postpartum hemorrhage – intervention frequency (%).


INTERVENTION	HOSPITAL – FREQUENCY (N = 4)

Analgesia	93%

Antibiotic therapy	93%

Bladder catheterization	70%

Oxytocin 40 units per liter over 6 hours	63%

Suturing of tissue trauma	52%

Uterine massage	48%

Misoprostol	41%

Tranexamic acid	37%

Laparotomy	11%

Manual removal of placenta	4%


### Midwifery Student Clinical Training

#### Preceptors

The level of preceptors varied, while ideally all would be midwives, there were many nurses. Of all the 39 surveyed, most preceptors (92%) reported being motivated by the opportunity to contribute to students’ training and professional development. When asked about the support preceptors sought, the majority reported a desire for increased financial compensation (56%), more training on how to precept (59%), and more access to clinical tools (67%). According to key informant interviews with maternity heads, although most preceptors in both CHCs and hospitals received training in teaching adults, little to no support was given beyond their initial preceptor training. Seventy-one percent of preceptors at CHCs and half of those at hospitals reported a need for more preceptors to support student learning.

Interviews with school staff, District Health Management Team (DHMT) staff, and heads of maternity (who were involved in patient care in addition to administrative duties), indicated that increased incentives for preceptorship would be appreciated and help surmount outside pressures; this could take the form of increased compensation, access to technology, or training.

Based on the data collected, there was no apparent process to oversee preceptors. Supervisory visits by the midwifery school and DHMT staff were not consistent and were often conducted concomitantly with student supervision. There was no standardization in how supervisors provided feedback or what follow-up was planned based on clinical observations. Eighty-five percent (85%) of preceptors surveyed at CHCs and 93% at hospitals reported it was challenging to balance the clinical workload with their teaching role. Some preceptors reported that abrupt shifts of staff to new facilities and scheduling issues have caused students to arrive at a site with no preceptor present.

#### Students’ pre-service education

Out of the 202 students surveyed, students reported inadequate learning opportunities in the following competency areas: assisting in the operating theater (42%), perineal suturing (32%), completing partographs (27%), and estimation of blood loss (23%). Students reported sufficient access to learning opportunities in the areas of medical history taking (65%), drug calculation and administration (62%), monitoring fetal and maternal well-being (56%), and fetal palpation (48%).

Qualitative data highlighted that preceptors assessed students through sign-offs on logbooks; however, this method may not have evaluated mastery. Several schools expressed concern that preceptors may not be providing honest feedback about student performance and competencies. In addition, student supervision via observation by school staff was often limited by lack of transportation and fuel costs. On the other hand, students were positive about the feedback they received from preceptors, most students reported receiving frequent feedback. Fifty percent of students reported receiving feedback daily, and 30% reported weekly feedback. However, only 40% of students reported preceptor feedback was specific and constructive. Some students reported arriving at CHCs only to have a Maternal and Child Health Aide for teaching, with no assigned preceptor. Of the 70% of students who reported attending a birth during their clinical placement, 52% attended or managed at least one delivery without a preceptor present.

#### Recent graduates

Out of the 74 recent graduates surveyed, some indicated they needed additional training in the management of sepsis (34%), post-abortion care (31%), assisted vaginal delivery (24%), and preeclampsia/eclampsia (22%). In their interviews, they expressed a desire for more continuing professional development and in-depth training opportunities, beyond the on-the-job training at the facility.

When asked about their confidence in managing specific skills independently, recently graduated midwives expressed feeling most confident in managing the following five areas independently: providing respectful maternity care (88%), Kangaroo Mother Care (86%), assessing fetal well-being (81%), active management of stage 1 and 2 of labor (78%), and monitoring pregnancy progression (76%).

### Infrastructure and resources

#### Drugs and consumables on labor wards

The four hospitals and four CHCs reviewed had limited availability of certain drugs. Dexamethasone, effective for lung maturation of premature infants, was only available 50% of the time in either hospitals or CHC’s and IV labetalol was not available at all. Furosemide was available 25% of the time in hospitals and 0% in CHCs. CHCs also did not have access to Hydralazine (Table 3).

**Table 3:** Drugs and consumables in hospital & community health centers.


DRUGS & CONSUMABLES	HOSPITAL N = 4	COMMUNITY HEALTH CLINIC N = 4

Magnesium sulfate	100%	100%

Oxytocin injectable	100%	75%

Oxytocin refrigeration	100%	100%

Misoprostol	100%	50%

Urinary catheters	75%	50%

Sutures in various sizes	75%	0%

Uterine balloon tamponade	75%	75%

Tranexamic acid	75%	0%

Ampicillin	75%	100%

Analgesia	50%	75%


##### Equipment and supplies

Although schools emphasized the importance of skills and simulation labs for student learning, students and preceptors voiced concerns that equipment and supplies in these labs did not reflect those in real-life clinical settings. The majority of stakeholders reflected that this reality made it difficult to apply their theoretical learning. One respondent shared that “… if they are taught the theory in school, the practical should be done in the facility, but the facility lacks all these necessary instruments.” Moreover, 50% of preceptors in CHCs and 42% of preceptors in hospitals felt strongly that their facility infrastructure and equipment were inadequate for teaching. A majority of respondents identified lack of infrastructure, equipment, and other resources as major challenges and specifically emphasized the following five areas: 1) Preceptor Lodging/Support (difficulty finding lodging, housing costs, and transportation costs), 2) Staffing Shortages (insufficient midwife staffing), 3) Equipment (shortages impacting student learning), 4) Facilities (unreliable electricity, water accessibility, space constraint), and 5) Medical Supplies (shortages of blood, PPE, delivery kits, drugs, pharmacy delays).

## Discussion

### Clinical care

Three factors have consistently played an important role in maternal deaths in low-income countries: delays in seeking care, delays in arriving at a health facility and inadequate care once at the facility [10,11]. Access to care is the first step towards receiving health services in any country. Studies have shown that Sierra Leonean women have not traditionally had access to or trust in health care facilities and providers [12,13]. Patients perceive hospitals as places to go only if pregnancy or delivery complications were likely. The women express concerns about incidental costs of medical care, inaccessibility of health facilities, doubts about the quality of care, and “disrespectful care” they might receive [12,13].

The findings of Tracey et al. and Oyerinde et al. are similar to this assessment and others who have studied why pregnant women in low-income countries, especially those living in rural areas, do not receive health services [12,13,14]. These factors include: a) paying for some parts of services even though universal government plans are available, b) physical inaccessibility of healthcare facilities, and c) delays or are prevented from receiving care.

Once patients arrive at a healthcare center in Sierra Leone, multiple challenges affect the care they receive: lack of partograph use for real-time clinical decision making, no standardization of clinical record keeping, and inconsistent and infrequent monitoring of vital signs. Our findings also revealed vague criteria for triaging and prioritizing care to women with immediate risks. Our results add to recent findings of Proos and colleagues who identified several onsite issues at Sierra Leone healthcare centers which affected care including communication and decision-making among health workers; women following up on the treatment suggestion; and logistical restrictions, including lack of equipment or medication or delays due to lack of ambulances [15].

Data from Sierra Leone’s 2017, 2018, and 2019 Maternal Death Surveillance and Response reports found that more than 80% of maternal deaths occurred at health facilities, and 75% of these mortalities were at government hospitals [16]. Postpartum hemorrhage (PPH) affects about 10% of all deliveries worldwide and it is the leading cause of maternal death among women, with 90% of these deaths occurring in low and middle-income countries [17]. Our results showed that 11% of women in hospital labor wards were diagnosed with postpartum hemorrhage, while only 1% of women at CHCs. This difference could be due to CHCs referring high-risk patients or having a limited ability to identify and diagnose postpartum hemorrhage. The team found that data around managing postpartum hemorrhage were often not reliable as the specific type of postpartum hemorrhage was frequently not well defined in the records, making it difficult to assess whether the type of intervention given was the most reasonable choice.

Timely and appropriately delivered emergency obstetric care (EmONC) by a skilled health care professional can lower maternal morbidity and mortality [18,19,20]. Yet, research in African countries has shown poor utilization of EmONC among women experiencing obstetrical complications, ranging from only 21% to 28% [21,22,23,24,25]. Our results showed that implementation of EmONC in Sierra Leone hospitals faces challenges, including: a) lack of protocols for management of obstructed labor, incomplete abortion, manual removal of placenta, ruptured uterus and; b) lack of essential medications such as hydralazine, furosemide, IV labelatol and dexamethasone. The WHO recommends uterine massage, uterotonics, and tranexamic acid as first-line interventions for PPH while second-line interventions include uterine balloon tamponade, uterine arterial embolization, and/or surgical intervention [10]. Unfortunately, our findings showed that 50% of CHCs were missing misoprostol and no CHCs had tranexamic acid. Women with postpartum hemorrhage did not receive bimanual compression nor uterine balloon tamponade. Suturing of tissue trauma, uterine massage, misoprostol use, and tranexamic acid are used less than 55% of the time based on available records; and CHCs did not have sutures available to repair lacerations.

All sites did post the EmONC protocols for postpartum hemorrhage, preeclampsia and eclampsia. Given that appropriate management of PPH requires early recognition and quick intervention, having trained staff with access to the necessary resources, and following proper protocols is critical [26].

### Preceptors and pre-service education

Our findings confirmed earlier work which identified barriers towards effective preceptorship such as inexperienced midwives/nurses, inadequate numbers of midwives, high turnover of midwives, heavy workloads, not enough time, lack of interest, poor remuneration, lack of personal reward for teaching, and increased student to preceptor ratios [27,28,29].

### Infrastructure and resources

CHCs are not currently equipped to treat postpartum hemorrhage with shortages of oxytocin, misoprostol, tranexamic acid, and other drugs. The shortages or unavailability of medical supplies such as blood, PPE, delivery kits and drugs; and the unreliability of electricity, water, and space all negatively affect the learning environment for students, preceptors, staff and patients.

## Limitations

While strengths of the present study include its context specificity and mixed approach, our findings are subject to several limitations. Data elicited from individual and focus-group interviews is inherently subject to a number of potential biases, including inaccuracy of interpretation and intentional or unintentional misleading (positive or negative skewing) by respondents. Our small sample size also frustrates any generalizability. Nevertheless, our data largely confirms previous research regarding the provision of maternal health care in Sierra Leone.

## Conclusions

The government of Sierra Leone and the Nursing and Midwives Board of Sierra Leone have initiated steps to address the identified issues in the needs assessment. While a lack of funding as well as physical and human resources have hindered steps forward, Sierra Leone has made progress in improving maternal care, substantially lowering its maternal mortality rate. Reducing maternal morbidity and mortality were critical objectives of the Reproductive, Newborn and Child Health Strategies of 2011–15 and 2017–21 produced by the MoHS. In partnership with the WHO, the MoHS produced the *Curriculum for Preceptorship Training Programme for Nursing and Midwifery*, which outlines a systems approach toward building clinical competency through improving the quality of academic and professional pre-service learning [7]. The Government has been strengthening its maternal care through initiatives and policy changes that include the *National Nursing and Midwifery Strategic plan 2019–2023* [6]. This template is the first comprehensive plan to address Sierra Leone’s nursing and midwifery challenges. The plan endorses three core features of Governance and Leadership, Education and Research, and Association Strengthening, and Partnerships, and specifically encourages increasing the skill and knowledge of nurses and midwives through education and better regulatory practices.

With national and international partners, the Nursing and Midwives Board of Sierra Leone developed the document, *Preceptorship Policy and Implementation Guidelines for Clinical Competency Building of Midwifery and Nursing Students and Residents* to address midwifery and nursing clinical education [7]. This policy acts as a national guide with standards for preceptors, educational institutions, and clinic facilities to deliver consistently high-quality experiences for students that lead to greater clinical competence upon graduation.

It was evident through the needs assessment that there are multiple challenges that impact the quality of clinical education in midwifery training programs. Poor collaboration between hospitals and health training institutions, a lack of clinical instructors and preceptors, and a weak framework for assessing students’ clinical performance remain a threat to quality education. Continued momentum and increased investments are required for quality clinical midwifery training, from consumables and equipment to record availability and accuracy. While this needs assessment highlights several obstacles for midwifery education and service delivery, there was a clear commitment across all participants to learning and strengthening maternal health outcomes.

## Appendix

### Sample of student survey questions on a 5-point Likert scale

1. I was satisfied with my clinical rotation and the opportunities to practice and develop my clinical skills.
2. The classroom modules helped prepare me for the clinical placement.
3. The time spent in the simulation lab helped prepare me for the clinical placement.
4. I had a strong understanding of the clinical competencies I was required to develop and practice before starting my clinical placement.
5. I felt supported by the School of Midwifery during my clinical placement.
6. On average, how many hours per day did you spend engaged in patient care at your clinical site?
7. My preceptor understood the academic elements of my degree program.
8. My preceptor treated me with respect.
9. My preceptor helped me improve my clinical skills.
10. My preceptor had strong teaching and mentorship skills.
11. How often did your preceptor provide feedback on your performance?
12. On average, how many hours per day did you spend with your preceptor?
13. Other than your preceptor, who provided additional support or mentorship during the placement?
14. How many births have you attended in your placements thus far?
15. Of those births, how many did you attend/manage without a preceptor or clinician present?

### Sample of focus group questions asked of preceptors

1. Why did you become a preceptor? Could you tell us more about your preceptor training?
2. Please walk us through your typical day as a preceptor. What do you do?
3. In your experience, what are the benefits of being a preceptor?
4. In your experience, what are the challenges of being a preceptor?
5. What types of support do you wish you had to be a more effective preceptor?
6. Have you ever participated in a training about mentorship, teaching or preceptorship? When? For how long? What topics were covered?
7. Could you tell us more about how you precept students? What strategies do you use?
8. How long do you work with each student? How do you provide feedback?
9. What do you think is working well for the training of student midwives at the clinical placements?
10. If you could design the clinical rotations and preceptor program for the next year of students at your facility, what would you change?
